# Evaluation of Alpha-Therapy with Radium-223-Dichloride in Castration Resistant Metastatic Prostate Cancer—the Role of Gamma Scintigraphy in Dosimetry and Pharmacokinetics

**DOI:** 10.3390/diagnostics5030358

**Published:** 2015-07-30

**Authors:** Kalevi Kairemo, Timo Joensuu, Nigora Rasulova, Timo Kiljunen, Aki Kangasmäki

**Affiliations:** 1 Departments of Molecular Radiotherapy & Nuclear Medicine, Docrates Cancer Center, Saukonpaadenranta 2, Helsinki FI-00180, Finland; 2 Medical Oncology, Docrates Cancer Center, Saukonpaadenranta 2, Helsinki FI-00180, Finland; E-Mail: timo.joensuu@docrates.com; 3 Radiotherapy, Docrates Cancer Center, Saukonpaadenranta 2, Helsinki FI-00180, Finland; E-Mails: timo.kiljunen@docrates.com (T.K.); aki.kangasmaki@docrates.com (A.K.)

**Keywords:** prostate cancer, alpha-emitters, radionuclide therapy, gamma imaging

## Abstract

Radium-223-dichloride (^223^RaCl_2_) is a new bone-seeking calcium analogue alpha-emitter, which has obtained marketing authorization for the treatment skeletal metastases of hormone-refractory prostate cancer. The current treatment regimen is based on six consecutive doses of ^223^RaCl_2_ at 4 week intervals and the administered activity dose, 50 kBq/kg per cycle is based on patient weight. We analyzed two patients using quantitative serial gamma imaging to estimate dosimetry in tumors and see possible pharmacokinetic differences in the treatment cycles. The lesions were rather well visualized in gamma scintigraphy in spite of low gamma activity (<1.1% gamma radiation) at 0, 7 and 28 days using 30–60 min acquisition times. Both our patients analyzed in serial gamma imagings, had two lesions in the gamma imaging field, the mean counts of the relative intensity varied from 27.8 to 36.5 (patient 1), and from 37.4 to 82.2 (patient 2). The half-lives varied from 1.8 days to 4.5 days during the six cycles (patient 1), and from 1.5 days to 3.6 days (patient 2), respectively. In the lesion half-lives calculated from the imaging the maximum difference between the treatment cycles in the same lesion was 2.0-fold (1.8 *vs*. 3.6). Of these patients, patient 1 demonstrated a serum PSA response, whereas there was no PSA response in patient 2. From our data, there were maximally up to 4.0-fold differences (62.1 *vs.* 246.6 ) between the relative absorbed radiation doses between patients as calculated from the quantitative standardized imaging to be delivered in only two lesions, and in the same lesion the maximum difference in the cycles was up to 2.3-fold (107.4 *vs.* 246.6). Our recommendation based on statistical simulation analysis, is serial measurement at days 0–8 at least 3 times, this improve the accuracy significantly to study the lesion activities, half-lives or calculated relative absorbed radiation doses as calculated from the imaging. Both our patients had originally two metastatic sites in the imaging field; the former patient demonstrated a serum PSA response and the latter demonstrated no PSA response. In these two patients there was no significant difference in the lesion activities, half-lives or calculated relative absorbed radiation doses as calculated from the quantitative imaging. Our results, although preliminary, suggest that dose monitoring can be included as a part of this treatment modality. On the other hand, from the absorbed radiation doses, the response cannot be predicted because with very similar doses, only the former patient responded.

## 1. Introduction

Prostate cancer is the most common cancer in Europe and 10%–20% of patients present with advanced or metastatic disease with associated problematic bone metastases [1]. Most castration-resistant prostate cancer (CRPC) patients have skeletal metastases. When the cancer establishes itself in the bone, the patient may feel pain, is more vulnerable to fractures and skeletal related events which may impair his health considerably. The main cause of disability and death among those with mCRPC is bone metastases.

Prior to radium-223 (Xofigo^®^, Bayer Healthcare AG, Berlin, Germany), in EU there were three registered radiopharmaceuticals available for bone pain palliation: Sr-89-Chloride (earlier Metastron^®^, generic Bio-Nucleonics, Miami, FL, USA), Sm-153-lexidronate (Quadramet^®^, CIS bio internationational, Gif-sur-Yvette, France) or Re-186-etidronate (Mallinckrodt, St Louis, MO, USA). These three radiopharmaceuticals are beta-emitters. According to EANM guidelines patients considered for ^89^Sr-Cl, ^153^Sm-EDTMP or ^186^Re-HEDP therapy had to fail in conventional analgesics and anti-tumor therapy, chemotherapy or hormone therapy. Pain had to be severe enough to limit normal activities and/or require regular analgesics and patients had to undergo recent (within 8 weeks or less) bone scintigraphy documenting increased osteoblastic activity at painful sites. These radionuclide treatments were palliative, where 60%–80% of patients benefit from the treatment and they did not cure metastatic cancer [2,3].

Radium-223 is the first bone-seeking radionuclide that is reported to increase overall survival, and quality of life by providing pain relief in their late stage disease from symptomatic skeletal events (SSEs) such as bone pain, pathological fractures, or spinal cord compression seen in up to 90% of mCRPC patients. This new drug, radium-223-dichloride (^223^RaCl_2_) is a new bone-seeking calcium analogue alpha-emitter, first of its kind in clinical use (Marketing authorization EU/1/13/873/001). It targets increased bone turnover developed by metastatic bone disease. In its double-blinded randomized registration trial the ^223^RaCl_2_ receiving patients demonstrated median overall survival of 14 months, *vs.* 11.2 months for those on placebo [4,5].

Radium-223 (^223^Ra) has a short-lived radon daughter (*i.e.*, ^219^Rn with t_1/2_ = 3.96 s).The decay chain is as follows: ^223^Ra (α, 11.4 d) ≥ ^219^Rn (α, 3.96 s) ≥ ^215^Po (α, 1.78 ms) ≥ ^211^Pb (β, 36.1 min) ≥ ^211^Bi (α, 2.17 min) ≥ ^207^Tl (β, 4.77 min) ≥ ^207^Pb (stable) [6,7]. Maximum α-energies of ^223^Ra deposited are 5.78, 6.88, 7.53 and 6.68 MeV and maximum β-energies deposited are 450 keV and 490 keV, respectively. Radium has five gamma energy peaks 82, 154, 269, 351 and 402 keV, which makes possible a relatively simple activity calibration and imaging (although low quality). The absorbed target doses in osteogenic cells are mainly delivered by alpha rays (1.140 Gy/MBq) when the total dose is 1.152 Gy/MBq (OLINDA) [3,8].

This Ra-223 is a bone targeting agent, at 10 min 12%, at 1 h 6% and at 24 h <1% of the injected activity is in the blood. The skeletal uptake was 44%–77% at 4 h. Faecal excretion is the major elimination route, but additionally 5% is excreted in the urinary tract [7].

The current ^223^RaCl_2_ dosing regimen is six cycles using 4 weeks intervals based on the ALSYMPCA (ALpharadin in SYMptomatic Prostate CAncer) (Alsympca) trial [4,5]. The administered activity will be a fixed dose base on patient weight, 50 kBq/kg.

In a new European legislation, including radionuclide therapy, dose planning will be a new prerequisite of radionuclide therapies (EC Directive 2013/59/Euratom) [9]. Therefore we present a method for relative dose calculation and therapy monitoring based on gamma imaging with therapeutic ^223^RaCl_2_ doses.

## 2. Methods and Case Histories

Radium-223-dichloride was used in our institution in patients with castration-resistant prostate cancer and oligometastatic skeletal disease using an activity dose 50 kBq/kg. The tracer was obtained from Bayer Pharma AG (Berlin, Germany). The current dosing regimen is six cycles with 4 weeks intervals.

**Figure 1:**
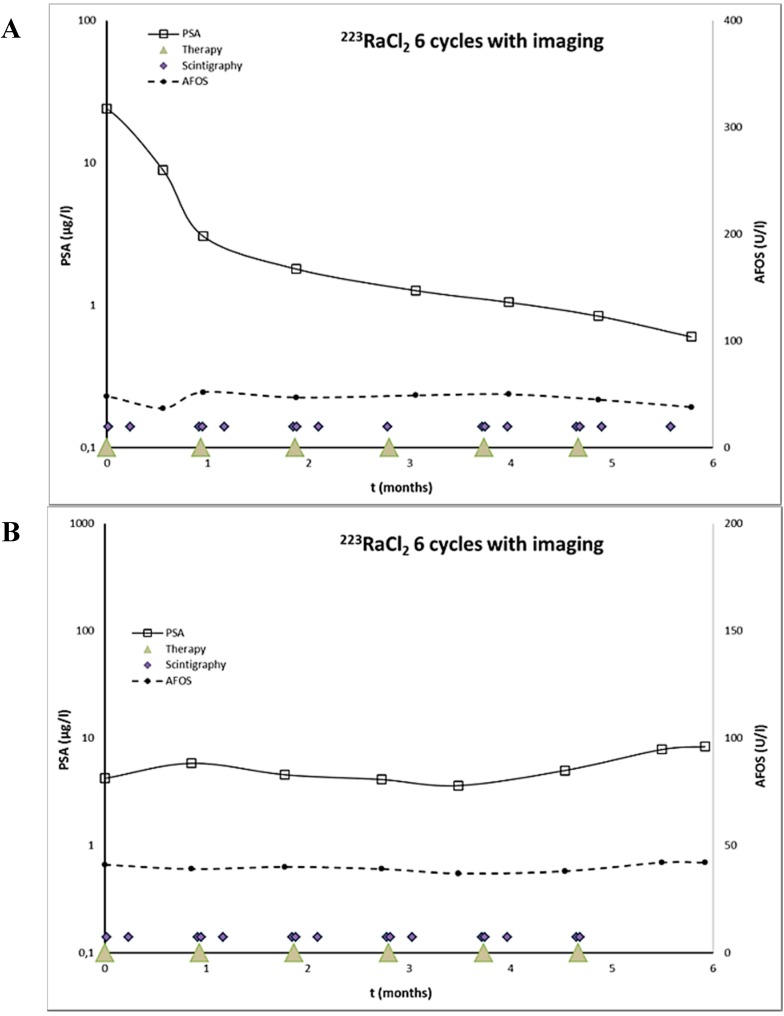
^223^RaCl_2_ therapeutic dosing schedule and timing of serial gamma camera imagings. S-PSA and S-AFOS oncentrations are presented as well. The patient 1 (**A**) demonstrated a S-PSA response, whereas the patient 2 (**B**) had an increasing S-PSA concentration. S-AFOS was almost unchanged in both patients during ^223^RaCl_2_ treatment courses.

Two of these patients were imaged with gamma camera after five cycles of in the six-cycle program within a 4-week interval. The patient 1 was imaged after first, second, fourth, fifth, and sixth cycle of radium-223-dichloride (Figure 1A), and the patient 2 after first, second, third, fourth and fifth cycle (Figure 1B). Both the patients gave their informed consent for all imagings, which were conducted in accordance with the Declaration of Helsinki and our patient database was approved by the Finnish authority for the protection of privacy and personal data.

Siemens Symbia gamma camera (Erlangen, Germany) equipped with medium energy collimator was used for gamma imaging at 0, 7 and 28 days after Ra-223-dichloride injection. The gamma energy peaks of 82, 154 and 269 keV were recorded using 15% energy windows, and summed to obtain better counting statistics. The acquisition time varied between 30 and 60 min per spot, depending on the time of last Ra-223 administration, in both AP and PA views. The imaging region in patient 1 was thoracic and in patient 2 the pelvic region. Both patients had two visually detectable metastatic sites in the imaging fields, based on both bone scintigraphy and fluorocholine-PET/CT imaging.

The tumor intensity was analyzed using ROI analysis; the background was an identical ROI on the contralateral site, and if the tumor was in the midline a region adjacent to it (with similar body dimensions) was used. The same ROIs were applied in all imaging studies during the same cycle. The tumor and background ROIs were applied to geometric mean images, calculated from geometric mean images. Differences in attenuation due to different thicknesses of patients and anatomical sites were not taken into account. The half-life was calculated by fitting a mono-exponential function sequentially and the remainder of activity due to earlier cycles was taken into account by extending each exponential fit beyond last imaging in order to see if there is a difference in the kinetic behavior between the cycles. The relative cumulated activity (relative to tumor dose) was calculated by multiplying the half-life in tumor with the relative intensity.

**Figure 2:**
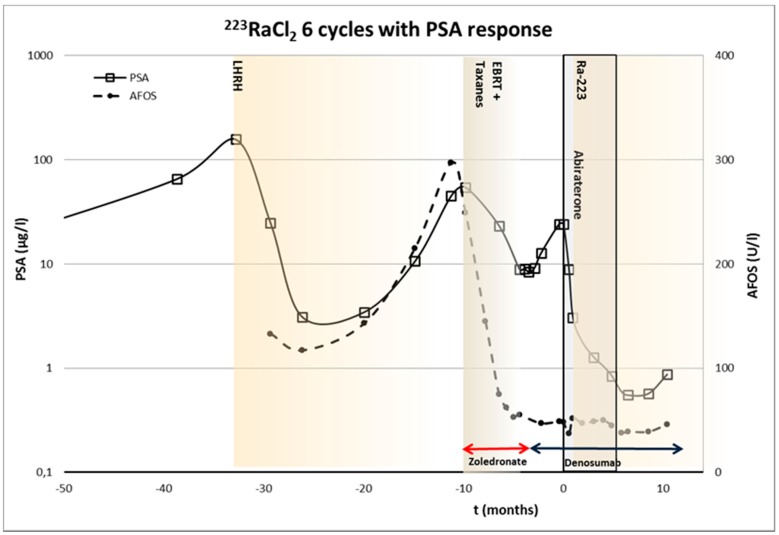
The timing of various treatments of 64-year-old male with prostate cancer (Gleason score 8 (4 + 4)), and ^223^RaCl_2_ therapeutic dosing schedule. S-PSA and S-AFOS concentrations are presented as well. This patient demonstrated a S-PSA response due to ^223^RaCl_2_ treatment.

Patient 1—Sixty-four year old male, biopsied Gleason score 8 (4 + 4), X/2010, S-PSA was 156. Skeletal metastases were seen on bone scintigraphy. LHRH treatment was started in X/2010 and combined with bicalutamide II/2011-V/2012. X/12 EBRT (20 Gy in four fractions) was given in upper thoracic and lower lumbar for salvage oligometastatic disease. XI/2012-II/2013 docetaxel was combined with zolendronic acid. VII/2013-XII/2013 ^223^RaCl_2_ was given. Abiraterone was started one week later. Before ^223^RaCl_2_, the S-PSA was 24.1 and I/2014 it was 0.60. The timing of various treatments is presented in Figure 2. This patient demonstrated a S-PSA response, *i.e.*, >50% decrease in S-PSA values.

This patient had two lesions in the gamma imaging field and further two lesions outside imaging field. The intensity of the initial relative intensity varied from 30.0 to 34.5 counts/pixel/hour (mean 33.0, SD 2.1) in the first lesion during the six cycles, and from 27.8 to 36.5 counts/pixel/hour (mean 30.9, SD 3.9) in the second lesion, respectively. The half-life in the first lesion varied from 1.8 days to 3.6 days (mean 2.4 days, SD 0.8 days) during the six cycles, and from 3.0 days to 4.5 days (mean 3.7 days, SD 0.6 days) in the second lesion, respectively. The relative calculated activity varied from 62.1 to 108.0 (mean 77.0, SD 20.9) in the first lesion during the six cycles, and from 100.5 to 125.1 (mean 113.6, SD 11.0) in the second lesion, respectively.

From this data, there were up to 2.0-fold differences (62.1 *vs.* 125.1) between the relative doses to be delivered in just two lesions, and in the same lesion the difference was up to 1.7-fold (62.1 *vs.* 108.0). It should be noted that variations in the half-life accounts for the most part of the relative dose differences.

**Figure 3:**
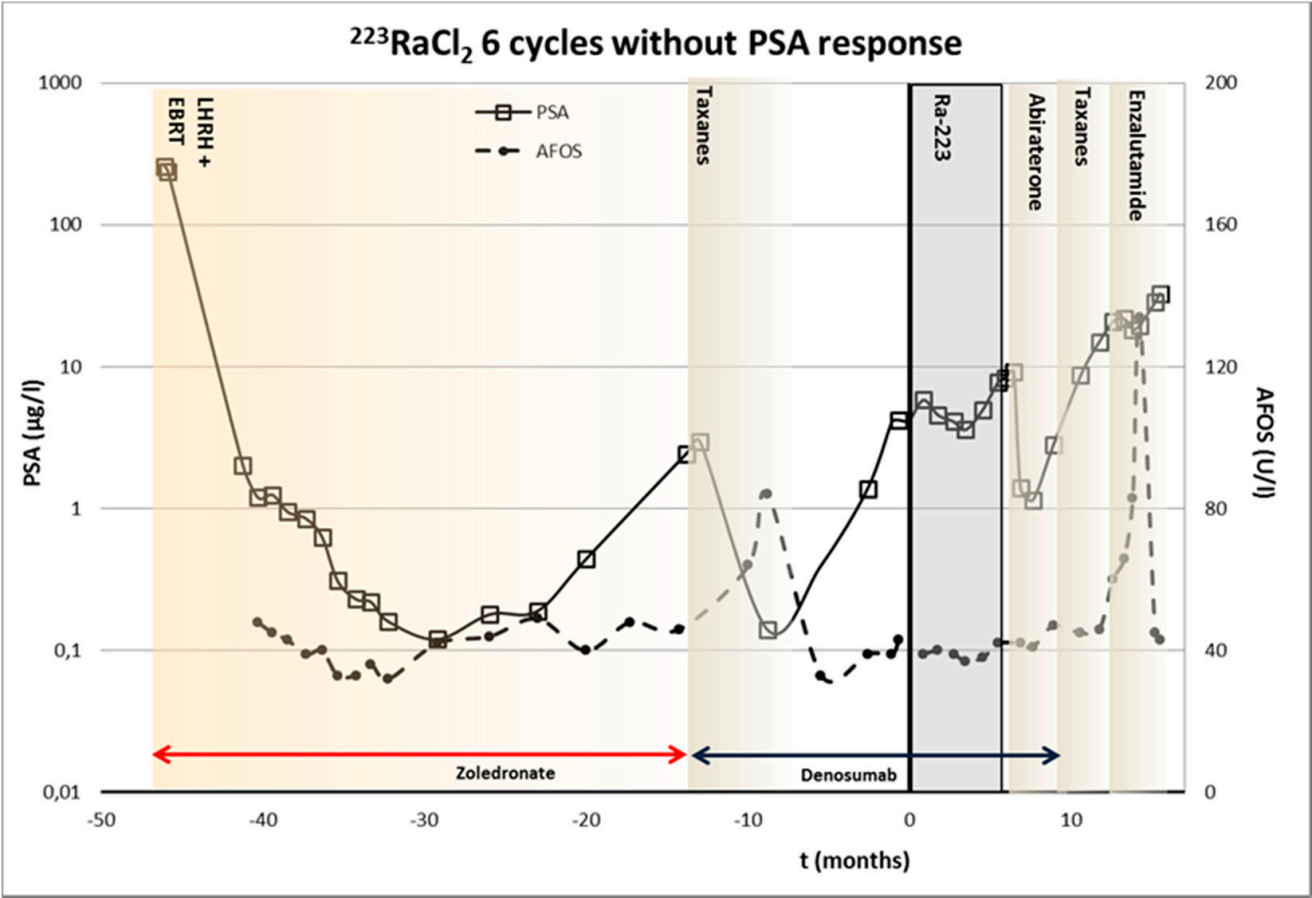
The timing of various treatments of 61-year-old male with prostate cancer (Gleason score 7 (4 + 3)), and ^223^RaCl_2_ therapeutic dosing schedule. S-PSA and S-AFOS concentrations are presented as well. This patient demonstrated only a change S-PSA velocity due to ^223^RaCl_2_ treatment.

Patient 2—Sixty-one year old male, biopsied Gleason score 7 (4 + 3), V/2009, S-PSA was 257. The staging was T_4_M_1_N_1_, including skeletal metastases in thoracic and lumbar spine, acetabulum, pubic bone, ischiadic bone, as well as left parailiac lymph nodes. He was treated originally with antiandrogens, LHRH analog and bisphosphonates. External beam radiation therapy was given in VII/2009 to prostate and left acetabulum. I/2012 docetaxel and denosumab were started due to progression. In II/2013-VII/2013 ^223^RaCl_2_ was given, but S-PSA was increasing, although with lower velocity as before. Abiraterone (androgen synthesis inhibitor) was started after ^223^RaCl_2_ it produced a short response, and new relapse was treated with taxotere and enzalutamide (androgen receptor signaling inhibitor). The timing of various treatments is presented in Figure 3. No S-PSA response was observed.

This patient had originally also two lesions, the intensity of the initial relative intensity was 37.4 in the first lesion during the 1st cycle, and it varied from 68.8 to 82.2 counts/pixel/hour (mean 74.0 SD 5.1) in the second lesion, respectively. The half-life in the first lesion 3.6 days during the 1st cycle, and it varied from 1.5 days to 3.0 days (mean 2.4 days, SD 0.6 days) in the second lesion, respectively. The relative dose was 134.6 in the first lesion during the 1st cycle, and it varied from 107.4 to 246.6 (mean 175.7, SD 49.7) in the second lesion, respectively. From this data, there were up to 2.3-fold differences between the doses in the same lesion (107.4 *vs.* 246.6). However, again the variation in the half-life accounts for the most part of the difference (up to 2-fold).

**Figure 4:**
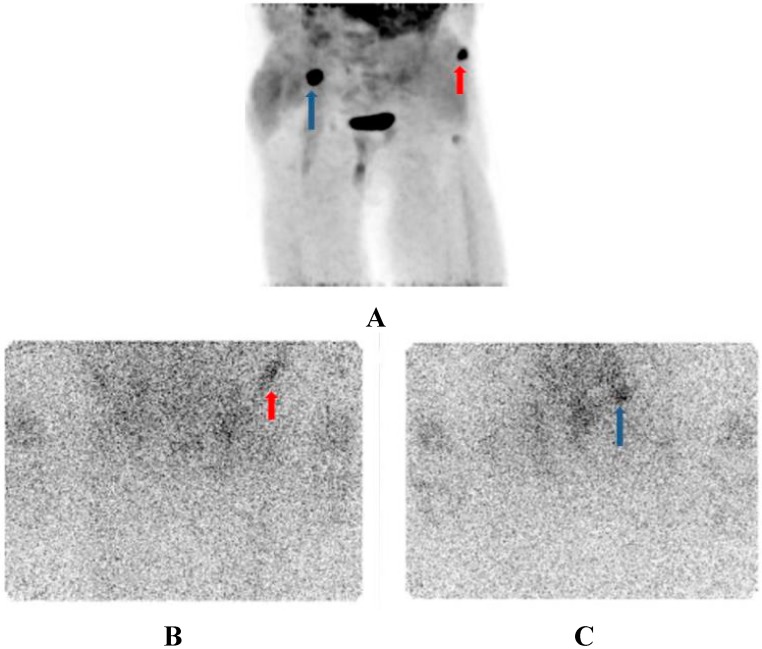
Diagnostic imaging with F-18-fluorocholine-PET; MIP-image (3-D-presentation); Strong uptakes in the right iliac crest (blue arrow) and left anterior ileal wing (red arrow) (**A**). Same lesion imaged at 7 days after ^223^RaCl_2_ therapeutic dose using gamma camera, the left anterior ileal wing (red arrow) lesion is better seen in the AP-view (**B**), whereas the lesion in the right iliac crest (blue arrow) is better seen in the AP-view (**C**).

The two measured lesions are shown in Figure 4.

In the Table 1 the gamma scintigraphy results are summarized. The relative intensities were more similar in patient 1. The lesions of the patient 2 are shown in Figure 4B,C. The lesion 1 demonstrated the lowest relative absorbed dose (corresponds the red arrow in the Figure 4A,B), whereas the lesion 2 in this patient got the highest relative absorbed dose (corresponds the blue arrow in the Figure 4A,C).

**Figure 5:**
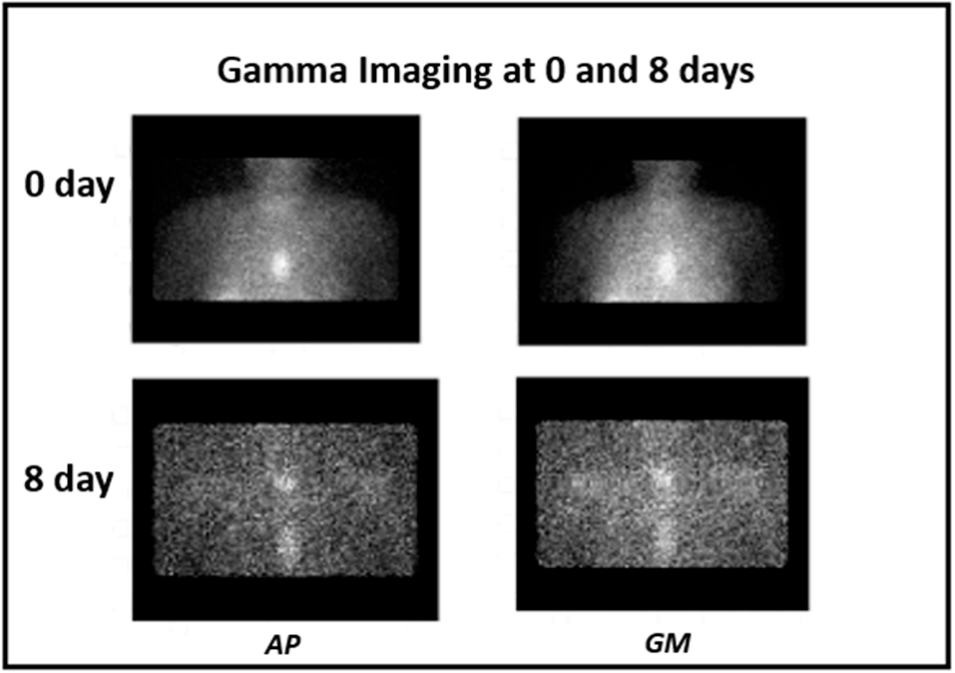
Gamma imagings at 0 and 8 days after ^223^RaCl_2_ therapeutic dose using gamma camera, AP-views on the left and geometric mean images on the right. This patient has a lesion in the sternum and in the thoracic vertebra.

The uptakes in the gamma images of the patient 1 are shown at 0 and 8 days after Ra-223 administration in Figure 5. The lesions in the midline are shown from AP-projections as well as geometric mean images.

**Table 1:** Summary of serial gamma imaging measurements (I-VI ^223^RaCl_2_ cycles, measurements on days 0, 7 and 28).

Patient/Lesion	Relative Intensity	Half-Life	Relative Absorbed Radiation Dose
Pt 1/1	33.0 ± 2.1 (30.0–34.5)	2.38 ± 0.83 (1.8–3.6)	77.0 ± 20.9 (62.1–108.0)
Pt 1/2	30.9 ± 3.9 (27.8–36.5)	3.73 ± 0.63 (3.0–4.5)	113.7 ± 11.0 (100.5–125.1)
Pt 2/1 *	37.4	3.6	134.6
Pt 2/2	74.0 ± 5.1 (68.8–82.2.9)	2.36 ± 0.56 (1.5–3.0)	175.7 ± 49.7 (107.4–246.6)

* one reliable reproducible measurement.

The reliability of the exponential fitting procedure for half-life calculations was assessed using Monte Carlo simulation of error propagation. Each of the lesion ROI’s and background ROI’s mean counts were used as Poisson distribution mean values. Sample size was the size of the respective ROI. 10,000 random numbers for all three imaging time points were generated, and Levenberg-Marquardt nonlinear fit was performed for all 10,000 time series generated. A_0_ and T_1/2_ values were fitted for every time series, and mean and standard deviations of the fitted parameters were calculated. This allowed us to assess the reliability of the A_0_ and T_1/2_ values. This approach does not take into account the fact that initial counting statistics were poor due to less than optimum imaging times. Choosing optimum imaging times, as stated above, will provide more reliable A_0_ and T_1/2_ data. We also know that medium energy collimators have about 10 mm resolution as compared to 0.1 mm range of alpha particles.

## 3. Discussion

There are now new therapies providing options to metastatic castration-resistant prostate cancer (mCRPC) patients, for whom just recently, therapies were ineffective and very limited based on docetaxel. These new therapies include androgen synthesis inhibitor (abiraterone) [10], androgen receptor signaling inhibitor (enzalutamide) [11], radionuclide therapy (radium-223) [12,13] and cabazitaxel chemotherapy [14].

Radium-223, is the first bone-seeking radionuclide that not only increases overall survival, but also increases quality of life [4,5]. In the Phase 3 ALSYMPCA (ALpharadin in SYMptomatic Prostate CAncer) registration trial [4,5], also all the secondary endpoints were also met, which included delay in time to first skeletal-related events: first events occurred in 13.6 months in the Ra-223 group as compared to 8.4 months in the placebo group—an improvement of 64%. 33% of ^223^RaCl_2_ patients had a total alkaline phosphatase normalization as compared to just 1% in the placebo group. Additionally, the ^223^RaCl_2_ patients had an improvement of 49% in time to PSA progression.

However, this ^223^RaCl_2_ is not an easy medication to use, because ^223^RaCl_2_ should be handled, delivered, received, used and administered only by authorized personnel in clinical settings. Their receipt, storage, use, transfer and disposal is regulated by EC directives and controlled national authorities, and special licenses for all procedures are required [15]. Both radiation safety and radiopharmaceutical quality criteria due to EU legislation have to be met. Gamma radiation (1.1%) allows imaging and convenient calibration of ^223^RaCl_2_. contamination can be measured using standard handheld surface contamination detectors for radioactivity. Urine, saliva, sweat, feces or vomiting of ^223^RaCl_2_ patients may cause a risk to health personnel or relatives. Sometimes maybe masks are necessary for risk groups in order not to inhale radioactivity. The only concern when dealing with alpha-emitters is internal contamination, because gamma radiation is negligible in posology. Gamma radiation (<1.1%) of ^223^RaCl_2_ may cause security alarms in some extremely sensitive detectors for radioactivity, these may be located at some airports, banks or border controls. Minimum required for authorized personnel in clinical settings is an experienced nuclear medicine department which has radionuclide therapy experience. They must have personnel (radiation safety officer (qualified person), nuclear medicine physician and technicians) who understand the nature of alpha-emitters, contamination and risk handling and who are capable for patient guidance [15]. Additionally, they must have good communication with referring physicians. The main side-effects should be managed easily and symptomatically, such as diarrhea [15].

Treatment response may be monitored, by using interim PET/CT studies, possible methods are Ga-68-PSMA-PET/CT or NaF-18-PET/CT, unfortunately there is not yet published data, but there are ongoing clinical trials. We present also here a method for monitoring response, because actually an additional lesion was observed in patient 2 as a sign of progression during the planned treatment course. However, the patient had no clinical symptoms and we continued with all planned six cycles of ^223^RaCl_2_.

This preliminary study where gamma scintigraphy was used to monitor pharmacokinetics and relative tumor dosimetry between treatment cycles turned out be possible to characterize lesions and they were well visualized in gamma scintigraphy. The acquisition times were rather long, 30–60 min. The lesion half-lives as calculated from the quantitative standardized imaging had large variation, the maximum difference between the treatment cycles in the same lesion was 2.0-fold (Table 1). Similarly, from our data, there were maximally up to 4.0-fold differences (62.1 *vs.* 246.6) between the relative absorbed radiation doses as calculated from the quantitative standardized imaging to be delivered in only two lesions (Table 1).

Because both our patients had originally two metastatic sites in the imaging field and because Patient 1 responded and Patient 2 demonstrated progression, imaging might give additional information about type of response or progression, especially if new lesions appear. Because patient 1 also got abiraterone, it is not possible to say how much the response here was dependent on Ra-223 or abiraterone or both. Indeed, any response in patient 1 is likely to be independent of Ra-223 given the lack of PSA response in patient 2 who received Ra-223 alone, and not in combination with abiraterone. In these two patients there was no significant difference in the lesion activities, half-lives or calculated relative absorbed radiation doses as calculated from the imaging. We have demonstrated after five cycles using measurements at 0 days, 7 days and 28 days, that there is up to 4-fold difference in the relative tumor doses between these cycles.

This suggests that dose planning and calculations should be included as a part of this treatment modality because lesion behavior is individual. On the other hand, from the absorbed radiation doses, the response cannot be predicted because with very similar doses, only the former patient responded.

Our method was rather simple, although time consuming: 30–60 min measurements at 0 days, ~7 days and ~28 days during six cycles of Ra-223. In a new European legislation, including radionuclide therapy, dose planning will be a new prerequisite of radionuclide therapies (EC Directive 2013/59/Euratom) [9]. This radiopharmaceutical product, Ra-223 is made with fixed concentration and the number cycles to be given may vary. This number can be calculated from administered 1st dose, and because we approximately know the variation between the cycles, our tool gives a preliminary answer to this task.

## References

[1] Wirth M., Horninger W. (2014). How I treat metastatic prostate cancer. J. OncoPathol..

[2] Bodei L., Lam M., Chiesa C., Flux G., Brans B., Chiti A., Giammarile F., European Association of Nuclear Medicine (EANM) (2008). EANM procedure guideline for treatment of refractory metastatic bone pain. Eur. J. Nucl. Med. Mol. Imaging.

[3] Pandit-Taskar N., Larson S.M., Carrasquillo J.A. (2014). Bone-Seeking radiopharmaceuticals for treatment of osseous metastases, part 1: α Therapy with ^223^Ra-dichloride. J. Nucl. Med..

[4] Sartor O., Coleman R., Nilsson S., Heinrich D., Helle S.I., O’Sullivan J.M., Fosså S.D., Chodacki A., Wiechno P., Logue J. (2014). Effect of radium-223 dichloride on symptomatic skeletal events in patients with castration-resistant prostate cancer and bone metastases: results from a phase 3, double-blind, randomised trial. Lancet Oncol..

[5] Parker C., Nilsson S., Heinrich D., Helle S.I., O’Sullivan J.M., Fosså S.D., Chodacki A., Wiechno P., Logue J., Seke M. (2013). Alpha emitter radium-223 and survival in metastatic prostate cancer. N. Engl. J. Med..

[6] Henriksen G., Fisher D.R., Roeske J.C., Bruland O.S., Larsen R.H. (2003). Targeting of osseous sites with α-emitting ^223^Ra: Comparison with β-emitter ^89^Sr in mice. J. Nucl. Med..

[7] Nilsson S., Larsen R.H., Fosså S.D., Balteskard L., Borch K.W., Westlin J.E., Salberg G., Bruland O.S. (2005). First clinical experience with alpha-emitting radium-223 in the treatment of skeletal metastases. Clin. Cancer Res..

[8] Deepika J. (2012). Radium-223 Dichloride: Bayer Responses to NRC Questions. http://pbadupws.nrc.gov/docs/ML1232/ML12320A450.pdf.

[9] Council Directive 2013/59/Euratom of 5 December 2013 laying down basic safety standards for protection against the dangers arising from exposure to ionising radiation, and repealing Directives 89/618/Euratom, 90/641/Euratom, 96/29/Euratom, 97/43/Euratom and 2003/122/Euratom. http://eur-lex.europa.eu/legal-content/EN/TXT/?uri=uriserv:OJ.L_.2014.013.01.0001.01.ENG.

[10] Logothetis C.J., Basch E., Molina A., Fizazi K., North S.A., Chi K.N., Jones R.J., Goodman O.B., Mainwaring P.N., Sternberg C.N. (2012). Effect of abiraterone acetate and prednisone compared with placebo and prednisone on pain control and skeletal-related events in patients with metastatic castration resistant prostate cancer: exploratory analysis of data from the COU-AA 301 randomised trial. Lancet Oncol..

[11] Scher H.I., Fizazi K., Saad F., Taplin M.E., Sternberg C.N., Miller K., de Wit R., Mulders P., Chi K.N., Shore N.D. (2012). Increased survival with enzalutamide in prostate cancer after chemotherapy. N. Engl. J. Med..

[12] Nilsson S., Strang P., Aksnes AK., Franzèn L., Olivier P., Pecking A., Staffurth J., Vasanthan S., Andersson C., Bruland Ø.S. (2012). A randomized, dose-response, multicenter phase II study of radium-223 chloride for the palliation of painful bone metastases in patients with castration-resistant prostate cancer. Eur. J. Cancer.

[13] Nilsson S., Franzén L., Parker C., Tyrrell C., Blom R., Tennvall J., Lennernäs B., Petersson U., Johannessen D.C., Sokal M. (2013). Two-Year survival follow-up of the randomized, double-blind, placebo-controlled phase II study of radium-223 chloride in patients with castration-resistant prostate cancer and bone metastases. Clin. Genitourin. Cancer.

[14] Basch E., Loblaw D.A., Oliver T.K., Carducci M., Chen R.C., Frame J.N., Garrels K., Hotte S., Kattan M.W., Raghavan D. (2014). Systemic therapy in men with metastatic castration-resistant prostate cancer:American Society of Clinical Oncology and Cancer Care Ontario clinical practice guideline. J. Clin. Oncol..

[15] Oyen W., Sundram F., Haug A.R., Kairemo K., Lewington V., Mäenpää H., Mortensen J., Mottaghy F., Virgolini I., O’Sullivan J.M. (2015). Radium-223 Dichloride (Ra-223) for the Treatment of Metastatic Castration-resistant Prostate Cancer: Optimizing Clinical Practice in Nuclear Medicine Center. J. OncoPathol..

